# Two-Dimensional Materials for Fabrication and Devices: Advances from Synthesis to Application

**DOI:** 10.3390/mi17020202

**Published:** 2026-02-02

**Authors:** Jiaqi Liu, Hu Long, Wu Shi

**Affiliations:** 1 State Key Laboratory of Surface Physics and Institute for Nanoelectronic Devices and Quantum Computing, Fudan University, Shanghai 200433, China; 19340190002@fudan.edu.cn; 2 Zhangjiang Fudan International Innovation Center, Fudan University, Shanghai 201210, China; 3 School of Mechanical Science & Engineering, Huazhong University of Science and Technology, Wuhan 430074, China; longgan88@hust.edu.cn

## 1. Introduction

Two-dimensional (2D) materials have rapidly emerged as a cornerstone of modern nanoscience, offering atomic-scale building blocks with extraordinary electronic, optical, and mechanical properties. Since the discovery of graphene in 2004 [1], the family of 2D materials has greatly expanded to include semiconducting transition metal dichalcogenides (TMDs) [2], black phosphorus [3], insulating dielectrics like hexagonal boron nitride (hBN), 2D metals [4], 2D carbides/nitrides known as MXenes, and even magnetic monolayers [5]. These atomically thin materials enable functionalities unattainable with traditional bulk structures. For example, disparate 2D layers can be stacked into van der Waals heterostructures on arbitrary substrates to create atomically sharp interfaces, yielding novel mechanical, electrical, magnetic, optical, and thermal behaviors that do not exist in the original materials [6,7,8]. Owing to such unique attributes, 2D materials are currently being explored for various applications ranging from nanoelectronics and spintronics to flexible electronics and sensing [9,10,11,12,13,14,15,16,17].

Equally important, however, are the practical challenges of transitioning 2D materials from the lab to robust device technologies. Many 2D materials (including graphene and certain TMDs) exhibit notable stability under ambient conditions, yet others—such as black phosphorus—degrade rapidly when exposed to air [18]. Even the “stable” 2D materials can face long-term reliability issues under varying environmental or operating conditions, an area that is not fully understood. Integration of 2D layers into functional devices also introduces new considerations. Interface engineering is required to ensure clean and well-adhered layer contacts, and high contact resistance at metal–2D junctions often limits device performance [19,20,21]. Strain effects in the flexible or layered 2D stacks can further modulate electronic and optical properties, sometimes adversely.

Researchers are beginning to tackle these challenges by developing better fabrication techniques and exploring novel 2D compounds. Advances in large-area synthesis (e.g., chemical vapor deposition) enable improved control over the number of layers, crystallinity, and heterostructure assembly [6,20,22]. At the same time, entirely new 2D materials (from 2D magnets to topological layers and MXenes) continue to be discovered, broadening the palette of functionalities available [23,24]. The convergence of materials innovation and device engineering has led to a surge of interest in deploying 2D materials in real-world technologies, from high-speed, low-power electronics to wearable devices, energy storage and harvesting systems, and even aerospace electronics [25]. Notably, recent studies show that certain 2D semiconductors can maintain unprecedented stability in extreme space environments, underscoring their promise for next-generation satellite and aerospace systems [26]. This Special Issue of *Micromachines*, entitled “2D-Materials Based Fabrication and Devices,” reflects the vibrant growth of this field. It gathers six contributions that span from fundamental studies of 2D material properties and novel material synthesis to advances in fabrication methods and device applications. In the following, we provide a thematic overview of these articles, grouping related works into three themes—(1) material synthesis and characterization, (2) interface engineering and integration techniques, and (3) 2D materials in sensing and device applications. We then discuss common challenges highlighted across these studies and outline future research directions for 2D material-based device technologies.

## 2. Thematic Overview

### 2.1. Material Synthesis and Characterization

The Special Issue features new developments in the 2D material family. Ma et al. report the synthesis and characterization of a novel 2D ternary TMD Ta_3_VSe_8_, using a chemical vapor transport (CVT) method [23]. Ternary TMDs (which incorporate two different metal elements) are relatively underexplored compared to binary TMDs, yet offer opportunities to tune properties via multi-element chemistry. In this work, high-quality Ta_3_VSe_8_ crystals are grown and confirmed by X-ray diffraction and electron microscopy to show its high crystalline quality and uniform composition. Temperature-dependent Raman spectroscopy (spanning from liquid nitrogen temperatures to room temperature) is also used to measure the intensity and position of Raman peaks. The results reveal that the peak intensity and positions are nearly unchanged across the whole temperature range, showing the stability of sample lattice and phonon mode; positive magnetoresistance is observed at low temperatures and gradually suppressed with the increase in temperature. This highlights the intriguing temperature-dependent electronic properties in this new material. By introducing Ta_3_VSe_8_, the authors open a possible avenue to broaden the group of available 2D TMD compositions and investigate phenomena beyond those observed in traditional binary TMDs. Expanding the library of 2D materials through such synthesis efforts is essential for discovering functionalities tailored to next-generation devices.

### 2.2. Interface Engineering and Integration

Several studies of this Special Issue aim to tackle the challenges of integrating 2D materials into device structures and improving the stability interfaces. Ievleva et al. explore the metastability of a van der Waals (vdW) interface between graphene and hBN through the transfer of graphene/hBN heterostructures onto microscale metal island arrays and subjecting them to repeated thermal cycling [27]. They observe that cooling from ambient to cryogenic temperatures and then reversing this causes irreversible changes in the electrical behavior of the device: the graphene–metal contacts degrade and signatures of suspended graphene regions vanish, coincident with the slight physical delamination of the 2D flake from the substrate. This instability is attributed to differential thermal expansion at the textured interface, which induces vdW bond breakdown and likely redistributes interfacial adsorbates. Interestingly, mild thermal annealing (“hot pressing”) can recover the graphene–metal contact, highlighting a possible route to re-stabilize the interface. These findings provide valuable insight into interfacial reliability—a critical issue for 2D material devices—and impose practical constraints on using transferred 2D heterostructures in environments with large temperature swings.

Achieving low-resistance, stable contacts to 2D materials is another key integration challenge addressed in this issue. Yang et al. invent a polycarbonate-assisted dry transfer method to form high-quality vdW contacts on air-sensitive magnetic 2D semiconductors [28]. Many magnetic 2D materials (e.g., CrSBr, Fe_3_GeTe_2_) are easily degraded in air and have insulating characters. This indicates that if a conventional lithograph method is used and metal is directly deposited on the surface of magnetic 2D materials, the contact resistance will be very high, which will hinder electrical transport measurement. The proposed PC-assisted technique allows electrodes to be laminated onto 2D flakes without exposure to any chemicals or air, thus preserving the pristine interface. Using this method, the authors achieved contact resistances several orders of magnitude lower than that of standard evaporated contacts in thin CrSBr devices. The approach also maintained the intrinsic magnetic properties of the 2D materials, enabling observation of sharp magnetic phase transitions in CrSBr that are comparable to those seen with ideal graphene contacts. Notably, the entire process is compatible with wafer-scale transfer and requires no additional lithography, offering a scalable solution for integrating fragile 2D magnets into spintronic devices. This contribution exemplifies how clever engineering of contact interfaces can overcome material-specific obstacles (like air sensitivity and poor conductivity) that currently limit the deployment of novel 2D materials.

In field of device component engineering, Bourahla et al. demonstrate hybrid material integration to enhance transparent conductive electrodes (TCEs) [29]. They present a high-performance electrode consisting of chemical-vapor-deposited (CVD) graphene layered on indium tin oxide (ITO) glass, doped with silver nanowires (Ag-NWs). The study addresses two integration challenges: residue removal from graphene transfer and uniform doping of the graphene film. A thermal annealing step is optimized to reduce polymer residues at the graphene–ITO interface, improving adhesion and electrical contact between graphene and the oxide. Subsequently, Ag nanowires are spin-coated onto the graphene to dope it, with controlled concentration and spin speed to ensure proper coverage. Through these steps, the hybrid TCE achieves a remarkably low sheet resistance of ~42 Ω/sq while maintaining ~87% optical transparency, outperforming either graphene or ITO alone. This significant improvement in the conductivity–transparency trade-off is attributed to the synergistic combination of materials: graphene provides excellent baseline conductivity and flexibility, ITO offers mechanical support and good transparency, and the Ag-NWs effectively dope the graphene to reduce its resistance. The result is a flexible, hybrid TCE suitable for touch screens, solar cells, and other optoelectronic devices that require both high electrical performance and transparency. More broadly, this work showcases how interface engineering (clean transfer, annealing) and material hybridization can be harnessed to push device components closer to practical requirements.

### 2.3. Sensing and Device Application

A recurring theme of this Special Issue is the application of 2D materials in advanced sensing technologies. Wang et al. develop a flexible, high-sensitivity strain sensor by combining a MXene with multi-walled carbon nanotubes (MWCNTs) in a self-assembled sliding network [30]. The MXene’s flakes, wrapped by dense multi-walled carbon nanotubes (MWCNTs), form an interpenetrating conductive network embedded in an elastic polymer. This design leverages the excellent conductivity of MXene and the long-range connectivity of MWCNTs to achieve both high gauge factor and large stretchability, typically a trade-off in strain sensors. The reported sensor in their research exhibits an impressive gauge factor of ~646 (indicative of high sensitivity) across a 40% strain range, along with a fast response time of 280 ms and the ability to detect tiny strain changes down to 0.05%. Such performance surpasses most conventional metal- or graphene-based strain sensors, especially in the field of flexible electronics. In a practical demonstration, the authors integrated these sensors into a wearable gesture-recognition glove. Using a trained convolutional neural network (CNN) to interpret the sensor signals, the glove could accurately distinguish 15 hand gestures with a recognition rate of 95%. This work illustrates the potential of 2D material composites in human–machine interfaces and wearable health/monitoring devices, where high sensitivity and durability under deformation are paramount.

Finally, Farina et al. highlight the use of graphene-based sensors for environmental monitoring in aerospace applications [25]. They report an ice detection sensor for aircraft that employs a thin graphene layer combined with a conductive polymer (PEDOT:PSS) to sense the formation of ice in real time. The sensor was systematically tested in a climatic chamber across various temperature and humidity conditions to simulate an aircraft flight envelope. The graphene-based device demonstrated enhanced sensitivity and rapid response to subtle environmental changes, especially around the critical water-to-ice phase transition. This acute sensitivity enables the detection of incipient ice formation, essentially catching the phase change at its onset, which is crucial for aviation safety. The authors report that the graphene/PEDOT:PSS sensor could reliably signal icing events before significant accumulation occurred, thanks to graphene’s conductivity changing with surface ice nucleation. Moreover, the sensor’s performance remained robust across repeated icing/de-icing cycles in the chamber. The study concludes that such 2D material-based ice sensors, being extremely lightweight, efficient, and responsive, could serve as superior alternatives to traditional ice detection technologies on aircraft. By providing earlier warnings of icing, they pave the way for improved in-flight safety and anti-icing control. This application exemplifies how 2D materials like graphene are moving beyond laboratories into specialized industries, addressing challenges (like icing) that demand new sensor capabilities.

## 3. Future Outlook

Collectively, the works in this Special Issue demonstrate both the progress and the ongoing challenges in 2D materials-based fabrication and devices. A common thread is the focus on stability and interface control, whether it is ensuring that a transferred 2D flake remains firmly bonded under thermal stress, preventing degradation of air-sensitive 2D layers during device assembly, or eliminating residue and contact resistance at material junctions. These studies highlight that mastering the 2D material interface (with substrates, electrodes, or other nanomaterials) is key to obtaining reliable device performance. Another shared theme is the integration of multiple materials or layers to overcome inherent limitations of a single material: for example, combining MXene with MWCNTs to achieve both high sensitivity and stretchability, or hybridizing graphene with ITO and Ag nanowires to balance conductivity and transparency. Such multi-material strategies are likely to become increasingly important as researchers tailor 2D material systems for complex device requirements.

Looking ahead, several directions emerge for future research. First, continued exploration of new 2D materials and heterostructures will expand the range of properties available for devices. The introduction of ternary TMDs like Ta_3_VSe_8_ is just one example; the further synthesis of multicomponent or metastable 2D phases (including oxides, nitrides, and MXene derivatives) could unlock phenomena beyond what is possible in current materials. Second, there is a need for developing scalable, contamination-free fabrication processes. Techniques such as the PC-assisted transfer for delicate 2D magnets point to the value of dry, residue-free assembly; extending such methods to broader materials and larger wafer scales will facilitate integration into commercial technologies. Similarly, improved transfer and encapsulation methods are needed to preserve 2D material quality (avoiding cracks, wrinkles, and polymer residues) when incorporating them into devices. Third, device-level innovations leveraging 2D materials should be pursued in real-world environments. The successes in wearable sensors and aviation icing sensors suggest that 2D materials can offer unmatched performance in some specialist field applications; expanding these concepts to other areas, such as biosensors, environmental monitors, or energy harvesters, would be a fruitful path. Field testing under operational conditions (as performed for the ice sensor) will be crucial for identifying failure modes and reliability issues early. Additionally, integrating 2D materials with traditional silicon microfabrication could bridge the gap between prototype demonstrations and manufacturable products. For example, incorporating 2D materials into CMOS-compatible processes, or using them in conjunction with MEMS technologies, could lead to hybrid devices that exploit the best of both worlds.

## 4. Conclusions

The research contributions in this Special Issue emphasize that 2D materials are moving from novel materials toward enabling components in diverse devices. They also remind us that achieving this transition requires not only discovering new materials and phenomena but also improving how we fabricate, combine, and stabilize these atomic layers in functional structures. We anticipate that the future research will build on these advances, delving deeper into the fundamental science of 2D interfaces and expanding the range of 2D material applications, ultimately driving the field toward practical technologies based on 2D materials.
